# Antibiotic Resistances and Molecular Characteristics of *Clostridioides difficile* in ICUs in a Teaching Hospital From Central South China

**DOI:** 10.3389/fmed.2021.745383

**Published:** 2021-12-06

**Authors:** Xiujuan Meng, Xun Huang, Zhong Peng, Yaowang Wang, Sidi Liu, Cui Zeng, Juping Duan, Ximao Wen, Chenchao Fu, Anhua Wu, Chunhui Li

**Affiliations:** ^1^Infection Control Center, Xiangya Hospital of Central South University, Changsha, China; ^2^State Key Laboratory of Agricultural Microbiology, College of Veterinary Medicine, Huazhong Agricultural University, Wuhan, China; ^3^National Clinical Research Center for Geriatric Disorders, Xiangya Hospital of Central South University, Changsha, China

**Keywords:** *Clostridioides difficile*, *Clostridioides difficile* infection, antibiotic resistance, molecular characteristics, ICUs, Central South China

## Abstract

*Clostridioides (C.) difficile* is a major healthcare-associated pathogen inducing infectious diarrhea. Approximately 25–33% of patients with antibiotic-associated diarrhea (AAD) and 90% of patients with pseudomembranous enteritis are caused by *C. difficile* infection (CDI). Stool samples were collected from hospitalized adults with presumptive AAD in four nonneonatal intensive care units (ICUs). Diagnosis of CDI was based on both clinical symptoms and laboratory results. The stool specimens were transferred onto CDIF (C. *difficile* agar), and *C. difficile* was finally confirmed by the latex agglutination test. Toxin-producing genes *tcdA* (A), *tcdB* (B), and *cdt* (CDT) were detected by PCR, and all isolates were performed multilocus sequence typing analysis. The antibiotic susceptibility of *C. difficile* isolates was assessed by the agar dilution method. A total of 184 *C. difficile* were isolated from 857 specimens in our study, the isolation rate of *C. difficile* was 21.5% (184/857). The 184 *C. difficile* were isolated from 179 patients, among these 115 patients were toxin-positive, giving the incidence of CDI being 58.0/10,000 patient days in the four ICUs. Among these 115 toxin-positive *C. difficile* isolates, 100 (87.0%) isolates produced two toxins (A+B+CDT-), three (2.6%) isolates were A+B+ with binary toxin-producing (A+B+CDT+), and 12 (10.4%) isolates only produced one toxin (A-B+CDT-). A total of 27 sequencing types (STs) were obtained. The most prevalent was ST3 (34 isolates), followed by ST39 (27 isolates), ST54 (19 isolates), ST26 (16 isolates), ST35 (15 isolates), and ST2 (13 isolates). All the ST26 isolates were nontoxigenic. Meanwhile, five STs were newly discovered. Although multidrug resistance was present in ≥50% of these *C. difficile* isolates, all of them were susceptible to tigecycline, fidaxomicin, metronidazole, and vancomycin. In conclusion, *C. difficile* isolates producing two toxins (A+B+CDT-) were dominant in our hospital. The most prevalent was ST3, and all ST26 isolates were NTCD. Although multidrug resistance was present in ≥50% of the *C. difficile* isolates, metronidazole, tigecycline, fidaxomicin, and vancomycin were still effective treatments for CDI in our hospital.

## Introduction

*Clostridioides difficile* is a major healthcare-associated pathogen inducing infectious diarrhea, which is responsible for a wide spectrum of diseases, ranging from mild diarrhea to fulminant colitis and even death (1). Approximately 25–33% of patients with antibiotic-associated diarrhea and 90% of patients with pseudomembranous enteritis are due to *C. difficile* infection (CDI) (2). The CDI has been associated with increased morbidity and decreased quality of life in patients, accompanied by prolonged hospitalization (3–5).

Recently, the incidence of CDI has been reported to increase in various countries. Sweden, China, and several other countries have reported an incidence rate of 17.1/10,000 admission to hospitals (6). The incidence of CDI has largely increased due to the emergence of an epidemic ribotype (sequence type [ST]: 1/027/NAP1), and the development of more sensitive detection approaches (7). CDI has been recognized to initiate from unusual antibiotic exposure of intestinal microbiota. The most important risk factor for CDI is broad-spectrum antimicrobial drugs that induce intestinal microfloral dysbiosis (8).

The severity and consequences of CDI are influenced by multiple factors, namely, hypervirulent isolates, age, immune status, and underlying conditions of the patient (e.g., receipt of antimicrobial therapy) (9). A novel *C. difficile* isolate with binary toxin-positive (non-027, non-078), associated with severe diarrhea has been recently reported in our hospital (10). Furthermore, the emergence of resistance to antimicrobial agents has complicated the treatment for CDI patients (11). Therefore, exploring the prevalence of antimicrobial-resistant *C. difficile* in an institution can facilitate optimizing antimicrobial stewardship programs. In this study, important information on the incidence of CDI was provided, and antibiotic resistance of *C. difficile* among patients in intensive care units (ICUs) wards was conducted.

## Materials and Methods

### Study Location and Population

A prospective study was performed to monitor patients from April 2017 to November 2017, identifying cases of hospital-onset diarrhea in four nonneonatal ICUs in Xiangya Hospital, which is a 3,500-bed tertiary university hospital in Changsha, Hunan Province, China, with approximately 100,000 annual admissions. The four nonneonatal ICUs include general ICU (GICU, 35 beds), neurosurgery ICU (NSICU, 20 beds), neurology ICU (NICU, 16 beds), and respiratory ICU (RICU, 10 beds). This study was approved by the Ethics Committee of Xiangya Hospital.

### Inclusion and Exclusion Criteria

#### Inclusion Criteria

Hospitalized patients aged ≥ 18 years old; diarrhea after 48 h following admission to the hospital; antibiotics administered before the occurrence of diarrhea (irregular stools ≥ 3 times per day with the Bristol grade as 5–7).

#### Exclusion Criteria

Hospitalized patients aged <18 years old; diarrhea within 48 h after admission to the hospital; irregular-shaped stools <3 times per day; patients with diarrhea diagnosed as gastrointestinal infection or intestinal functional diseases.

### Isolation of *C. difficile* and CDI Diagnosis

Stool specimen was collected from the patients with diarrhea occurring ≥ 48 h after admission and before discharge, and specimen (about 1 g) was taken and placed in 1 ml of sterile saline and mixed well, then transferred onto CDIF agar (Chrome ID *C. difficile*) (Biomerieux, Shanghai, China) and incubated in anaerobic airtight containers (Biomerieux, Shanghai, China) for ≥ 48 h. *C. difficile* isolates were identified by odor and colony morphology, followed by final confirmation with latex agglutination test using glutamate dehydrogenase (Biomerieux, Shanghai, China) and PRO DISK (Remel, England).

The 16S rDNA and toxin-producing genes *tcdA, tcdB, cdtA*, and *cdtB* were conducted by PCR according to prior recommendations (12). 16S rDNA was an internal positive control in toxin gene PCR. The patients with stool cultured positive for *C. difficile*, meanwhile, the isolates that tested positive for toxin gene by PCR were diagnosed with CDI.

### MLST Analysis of *C. difficile*

Multilocus sequence typing (MLST) was performed to analyze the *C. difficile* isolates (both toxigenic and nontoxigenic) following a previously established method (13). The genomic DNA was obtained from *C. difficile* cultured on blood agar (BioMerieux, Shanghai, China) for 48 h at 37°C under anaerobic conditions. High molecular weight DNA extracted using QIAamp DNA Mini Kit (QIAGEN, Valencia, CA, USA) according to the instructions of the manufacturer. The specimen was coded with a unique study ID, collection dates, test results, and the data of the patient were registered.

### Determination of Antibiotic Resistance

The susceptibility of *C. difficile* isolates to 11 types of antibiotics, namely, chloramphenicol (CHL), metronidazole (MTZ), vancomycin (VAN), rifaximin (RFX), fidaxomicin (FDX), ampicillin (AMP), clindamycin (CLI), tigecycline, fusidic acid (FSA), levofloxacin (LVX), and tetracycline (TE) were tested by agar dilution according to the procedures of the Clinical and Laboratory Standards Institute (CLSI-M100-S29). The minimum inhibitory concentrations (MICs) defined as the lowest concentration of each antimicrobial agent that inhibited the growth of the tested isolate, were recorded after 48 h of incubation following CLSI recommendations. The antibiotics were purchased from MedChemExpress (America).

MIC50 and MIC90 referred to the MIC required to inhibit the growth of 50% and 90% of the tested bacteria. *C. difficile* ATCC 70057 and *Bacteroides fragilis* ATCC 25285 were used for quality control. The interpretive breakpoints for CHL (MIC ≥ 32), CLI (MIC ≥ 8), TE (MIC ≥ 16), AMP (MIC ≥ 2), LVX (MIC ≥ 8), and MTZ (MIC ≥ 32) were set following the guidelines recommended by CLSI (https://clsi.org/media/1872/_m100_archived_drugs_table.pdf). The breakpoint for VAN (MIC > 2) was based on the European Antimicrobial Susceptibility Test Committee (14). The resistance breaking points for RFX (MIC > 32) and FSA (MIC > 0.5) were according to the literature (15). No resistance breaking points were available for fidaxomicin and tigecycline with specific.

### Statistical Analysis

Data were presented by the rate, and the comparison of antibiotic resistance rate was conducted by chi-squared test. *P* < 0.05 indicates that the difference is statistically significant.

## Results

### *C. difficile* Isolation and Isolation Rate

**Figure 1:**
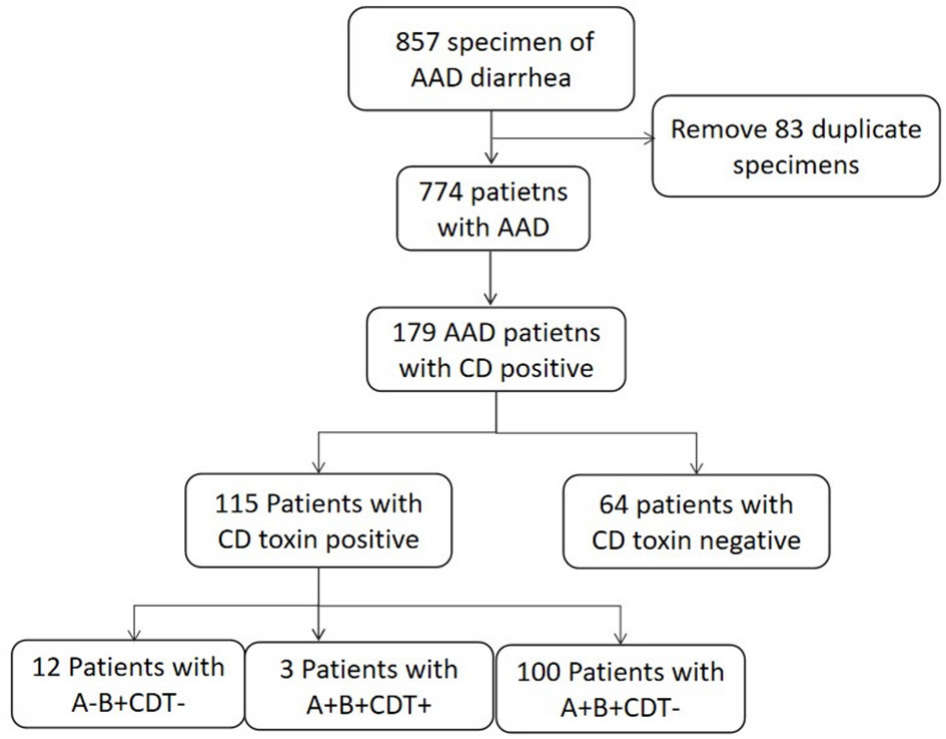
The detailed procedures of patient enrollment and specimen screening processes.

A total of 857 specimens meeting the criteria were collected, among which 184 *C. difficile* were isolated, with an isolation rate of *C. difficile* as 21.5% (184/857). Repetitive specimens from the same patient were excluded. Among the total 774 patients with diarrhea, 179 patients were positive for *C. difficile* culture, while the other 595 patients were *C. difficile* negative (Figure 1).

### Incidence of CDI in Four ICUs

Among the 179 patients, 115 patients (14.8%, 115/774) tested positive for toxin production and were diagnosed with CDI. Of the 115 patients, 12 patients were with A-B+CDT-, 100 patients were with A+B+CDT-, and three patients were with A+B+CDT+. The incidence of CDI in the patients from four ICUs was 58.0/10,000 patient days. Among these CDI patients, 70 patients were from GICU (82.0/10,000 patient days), 31 patients were from NICU (75.7/10,000 patient days), seven patients were from NSICU (15.3/10,000 patient days), and seven patients were from RICU (26.4/10,000 patient days). The incidence rate of CDI among four ICUs departments were shown in Table 1.

**Table 1:** The incidence rate of *C. difficile* infection among four ICUs departments.

	**Total patients (cases)**	**Diarrhea patients (cases)**	**CD toxin-positive patients (cases)**	**Patient days**	**Incidence rate of *CDI* (/10,000 patient days)**
GICU	620	558	70	8,537	82.0
NICU	153	148	31	4,095	75.7
NSICU	40	33	7	4,565	15.3
RICU	44	35	7	2,647	26.4
Total	857	774	115	19,844	58.0

*CDI incidence calculated as C. difficile toxin-positive patients/patient days*.

### Clinical Features and Antibiotics Exposure of CDI Patients

The patients with CDI were mostly men (78 patients), and 39 patients were ≥ 65 years old, the common underlying diseases were hypertension, diabetes, and consciousness disorder in these patients. Among these 115 patients with CDI, 35 (30.43%) patients used two or more antibiotics in combination. Meanwhile, 32 (27.83%) patients used carbapenems, 31 (26.95%) patients used β-lactam mixture, and 29 (25.21%) patients used third and fourth generation cephalosporins. The clinical features and antibiotics exposure of CDI patients were shown in Table 2.

**Table 2:** Clinical features and antibiotics exposure of the CDI patients.

**Variable**	**Case (*n* = 115)**	**Antibiotics exposure**	**Case (*n* = 115)**
Age ≥ 65 year	39 (33.9%)	Two kinds and more	35 (30.4%)
Sex, male (%)	78 (67.8%)	Cephamycins	18 (15.7%)
Diabetes	23 (20.0%)	Cephalosporin (3th and 4th)	29 (25.2%)
Hypertension	43 (37.4%)	Cephalosporin (1th and 2th)	4 (3.5%)
Respiratory failure	8 (7.0%)	β-lactam mixture	31 (26.9%)
Renal insufficiency	10 (8.7%)	Tigecycline	5 (4.4%)
Cardiac insufficiency	12 (10.4%)	Carbapenems	32 (27.8%)
Tuberculosis	4 (3.5%)	Penicillins	25 (21.7%)
Tumor	11 (9.6%)	Glycopeptides	10 (8.7%)
Autoimmune diseases	3 (2.6%)	Linezolid	3 (2.6%)
Liver disease	14 (12.2%)	Quinolones	14 (12.2%)
Consciousness disorder	25 (21.7%)	Antifungal	8 (7.0%)

### MLST Analysis of *C. difficile* Isolates

A total of 27 STs were identified in the above-mentioned 179 *C. difficile* strains, which were divided into four main clades. Among these STs, the most prevalent types were ST3 (34, 19.0%), followed by ST39 (27, 15.1%), ST54 (19, 10.6%), ST26 (16, 8.9%), ST35 (15, 8.4%), ST2 (13, 7.3%), ST37 (9,5.0%), ST129 (7, 3.9%), ST15 (5, 2.8%), ST83 (5, 2.8%), ST5 (3, 1.7%), ST14 (3, 1.7%), ST33 (3, 1.7%), ST476 (2, 1.1%), and one isolate for each of the other STs. All the ST26 isolates were nontoxin-producing *C. difficile*. Moreover, five STs were newly discovered, among which ST475 was obtained from SICU, ST476 was from both SICU and SGICU, while ST477, ST478, and ST479 were from GICU. Meanwhile, only one gene (*tpi)* was found different between ST477 and ST37 isolates. The novel ST data have been submitted to the MLST database.

### Toxin Gene Detection of *C. difficile* Isolates

**Figure 2:**
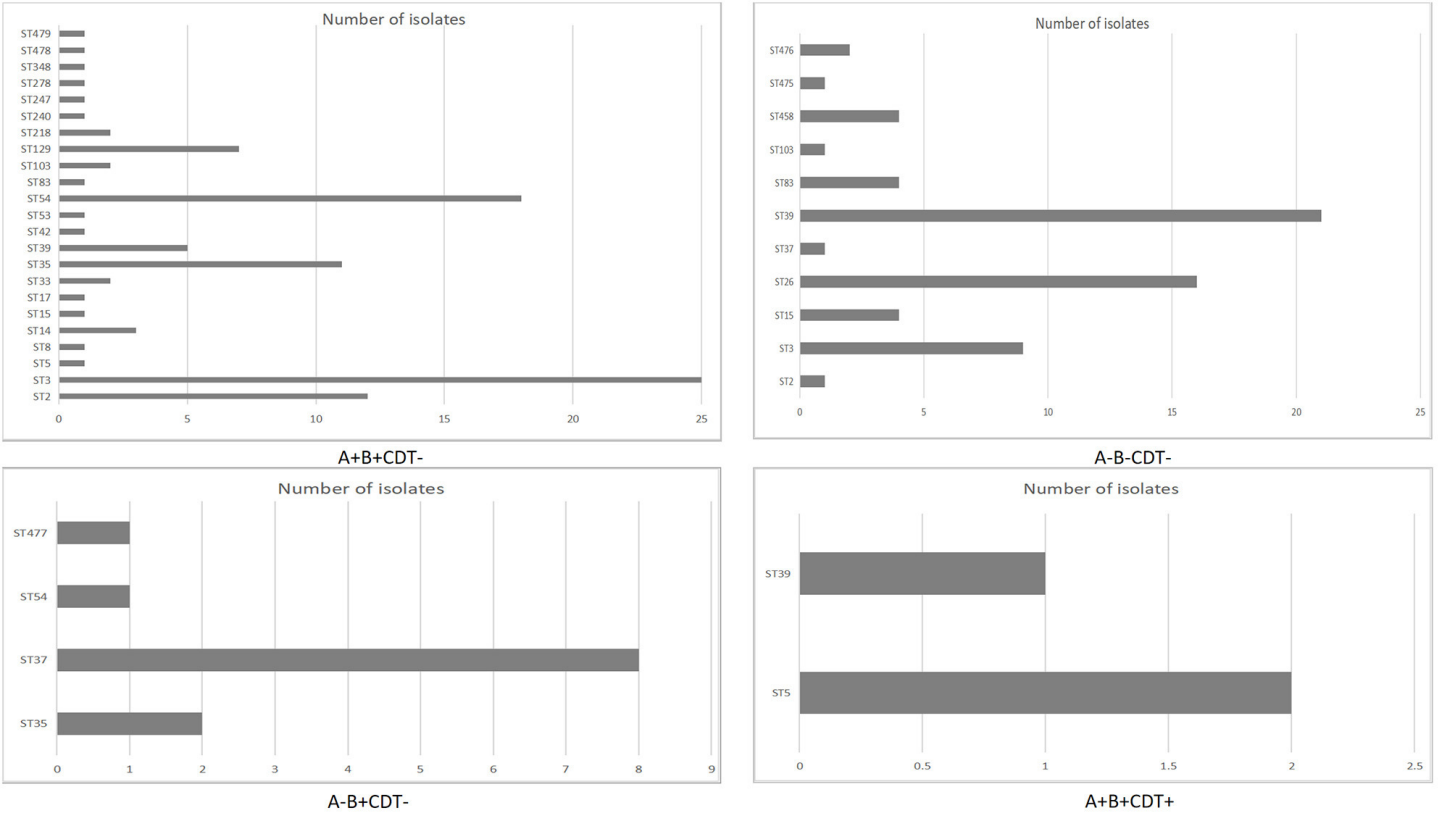
The relationship of sequence types and toxin genes of *C. difficile* isolates.

A total of 179 nonrepetitive *C. difficile* isolates were collected in this study, 115 isolates (64.2%, 115/179) were toxin-producing, while the rest 64 isolates (35.8%, 64/179) were nontoxigenic, and ST26 were all nontoxigenic isolates. Among these 115 toxin-producing isolates, 100 isolates (87.0%, 100/115) were positive for the two toxin genes (tcdA and tcdB), but negative for binary toxin encoding genes (A+B+CDT-); three isolates (2.6%, 3/115) were positive for both tcdA and tcdB, and the binary toxin encoding genes (A+B+CDT+), which belonged to ST5 and ST3; 12 isolates (10.4%, 12/115) could produce only tcdB, but negative for tcdA and binary toxin genes (A-B+CDT-). Among the *C. difficile* isolates with two toxin genes (A+B+CDT-), ST3 and ST54 were the main ST types. *C. difficile* isolates with both two toxin genes and binary (A+B+CDT+) belonged to ST5 and ST39, and ST37 were the most common ST type in *C. difficile* isolates with only tcdB (A-B+CDT-). All the ST26 isolates in this study were nontoxigenic (NTCD). The relationship of sequence types and toxin genes of *C. difficile* isolates was shown in Figure 2.

### Antibiotic Resistance Rate Among Toxigenic *C. difficile* Strains

#### Antibiotic Resistance Rate of *C. difficile* Isolates According to Toxin Type

Multidrug resistance was present in *C. difficile* isolates in this study, while no significant difference was observed between isolates with A+B+CDT- and those with A-B+CDT- (Table 3). The most prevalent resistance was detected for CLI (81.0 and 91.2%) and CHL (89.0 and 91.2%), followed by AMP (68.0 and 50.0%) and TE (50.0 and 50.0%). Meanwhile, the resistance to LVX (47.0 and 50.0%) and FSA (44.0 and 41.7%) was also high. The MIC values of CHL (MIC90 ≥ 256 μg/ml), CLI (MIC90 ≥ 128 μg/ml), TE (MIC90 ≥ 128 μg/ml), AMP (MIC90 ≥ 128 μg/ml), and LVX (MIC90 > 128 μg/ml) were relatively high, while those of tigecycline (MIC90 = 0.25 μg/ml) and fidaxomicin (MIC90 = 0.5 μg/ml) were relatively low. Moreover, we found that all the 115 isolates were susceptible to MTZ and vancomycin.

**Table 3:** The antibiotic resistance of *C. difficile* isolates according to toxin type.

	**tcdA+** **tcdB+ctdA-ctdB-(*****n*** **=** **100 isolates)**				**tcdA- tcdB+ctdA-ctdB-(*****n*** **=** **12 isolates)**				***P* value**
	**Range**	**MIC 50**	**MIC 90**	**Resistance rate (%)**	**Range**	**MIC 50**	**MIC 90**	**Resistance rate (%)**	
MTZ	0.125–0.5	0.25	0.5	0	0.125–0.5	0.25	0.5	0	-
VAN	0.125–2	0.5	1	0	0.25–1	0.5	1	0	-
TGC	0.125–0.5	0.25	0.25	-	0.125–0.25	0.25	0.25	-	-
CLI	0.125–256	32	128	81.0	0.5–256	64	128	91.2	0.36
CHL	0.25–256	256	256	89.0	4–256	256	256	91.2	0.78
TE	0.125–256	16	256	50.0	0.25–256	4	128	50.0	1.00
AMP	0.25–256	2	128	68.0	0.25–256	2	128	50.0	0.21
LVX	0.25–256	4	256	47.0	0.25–256	4	128	50.0	0.84
RFX	0.125–256	0.25	8	5.0	0.25–128	0.25	0.25	16.7	0.12
FSA	0.125–64	0.5	8	44.0	0.25–8	0.25	2	41.7	0.87
FDX	0.125–0.5	0.25	0.5	-	0.125–0.5	0.25	0.5	-	-

#### Antibiotic Resistance Rate of *C. difficile* Isolates According to ST Type

A comparison of the prevalence of antibiotic resistance among different STs of the *C. difficile* isolates was shown in Table 4. The rates of CLI (58.3%), TE (33.3%), and AMP (50%) resistance in ST2 *C. difficile* isolates were lower than those in other STs. Meanwhile, the prevalence of CHL resistance was high in all STs (66.7–100%), while a great variety was observed in that of FSA (0.0–73.6%). In addition, most of the *C. difficile* isolates were sensitive to rifaximin (0.0–16.7%).

**Table 4:** The antibiotic resistance of *C. difficile* isolates according to ST types.

**ST (strains)**	**CHL (%)**	**CLI (%)**	**TE (%)**	**AMP (%)**	**LVX (%)**	**RFX (%)**	**FSA (%)**
ST2 (*n* = 12)	100.0	58.3	33.3	50.0	83.3	8.3	33.3
ST3 (*n* = 25)	88.0	80.0	36.0	56.0	52.0	0.0	16.0
ST35 (*n* = 13)	76.9	92.3	61.5	69.2	15.3	7.7	23.1
ST37 (*n* = 8)	87.5	87.5	50.0	62.5	62.5	12.5	37.5
ST39 (*n* = 6)	66.7	83.3	83.3	66.7	83.3	16.7	50.0
ST54 (*n* = 19)	84.2	84.2	42.1	68.4	42.1	0.0	73.6
Others (*n* = 32)	100.0	84.4	62.5	78.1	37.5	9.4	0.0

#### Antibiotic Resistance Type of Toxigenic *C. difficile* Isolates

**Figure 3:**
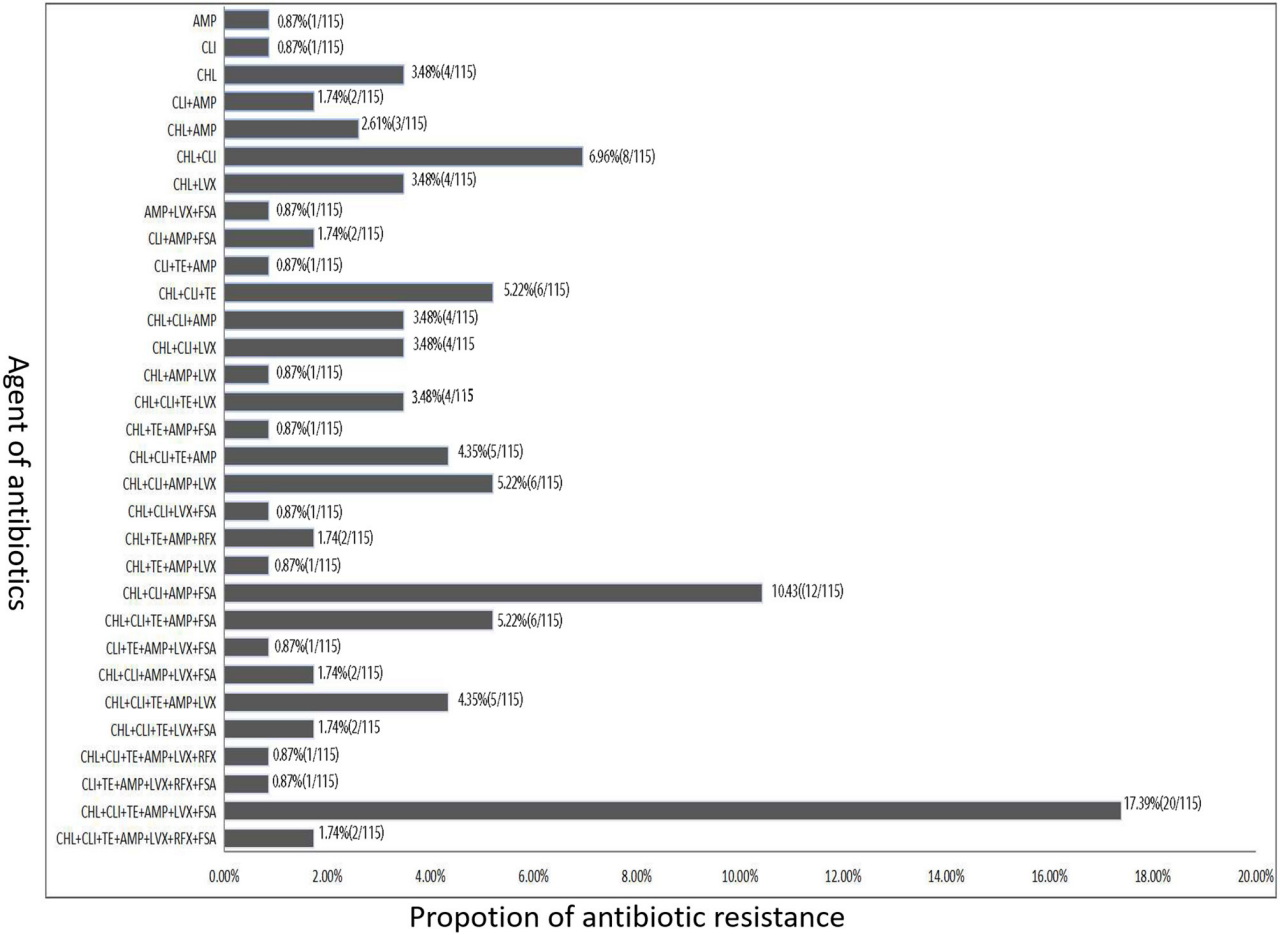
The antibiotic resistance type of toxigenic *C. difficile* isolates according to the difference of resistance to different types of antibiotics.

Except for one *C. difficile* isolates was sensitive to all the seven antibacterial drugs in our study, other isolates were found to be resistant to at least one of these antibiotics, and most of them were resistant to either CHL, CLI, or AMP (Figure 3). Among these 115 toxigenic *C. difficile* isolates, 92 isolates (80.0%) were resistant to at least three antibiotics, and multiple resistance to CHL, CLI, TE, AMP, LVX, and FSA (20 isolates) were most common, followed by 12 isolates were resistant to four antibiotics (CHL+CLI+AMP+FSA), six isolates were resistant to another four antibiotics (CHL+CLI+AMP+LVX), and another six isolates were resistant to three antibiotics (CHL+CLI+TE). A total of 72 isolates were resistant to more than three antibiotics. Among these isolates, 32 isolates were resistant to four antibiotics, 13 isolates were resistant to five antibiotics, 22 isolates were resistant to six antibiotics, and only two isolates were resistant to the seven antibiotics.

## Discussion

Patients with diarrhea are not routinely tested for *C. difficile* in China, therefore, CDI might be underestimated (16–18). In our study, we actively tested patients in the ICU ward, the isolation rate of *C. difficile* was 21.5% (184/857), which is similar to the study by Polage et al. (19), in which 21.0% (293 out of 1,416) of hospitalized adults were tested *C. difficile* positive by PCR. However, another study with an immunochromatographic test reported a 4.9% positive rate among the patients with diarrhea (20), which is much lower than our results. The difference might be due to the limitations and methods of the studies, or might be related to the size of the study population. Moreover, the ICU patients in our study almost received broad-spectrum antibiotics, which might have potentially induced the emergence of CDI (21).

The positive rate (14.8%, 115/774) of *C. difficile* toxin genes in this study is higher than our previous study (8%, 47/593) (9). The overall incidence rate of CDI (58.0/10,000 patient days) is also higher than our previous study (14.1/10,000 patient days) (4), and the highest incidence of CDI was found in GICU patients (82.0/10,000 patient days). This might be due to the wide administration of broad-spectrum antibiotics in recent years. In our study, among these 115 patients with CDI, 35 patients used two or more antibiotics in combination. Meanwhile, 32 patients used carbapenems, 31 patients used β-lactam mixture. In addition, the higher positive rate might be related to the use of the chromogenic medium, which could better detect *C. difficile*. A study by 17 hospitals in South Korea found that the CDI incidence increased from 17.0/100,000 adult inpatients in 2004 to 27.0/100,000 adult inpatients in 2008 (22). Moreover, the incidence rate reported in the United States during the same period was 87.5/100,000 hospitalized patients (23). The reasons for the increased incidence rate might be related to the advanced detection approaches.

Although A+B+ (87.0%) was the dominant *C. difficile* toxigenic type found in this study, the prevalence of A-B+ has been reported to increase in several other regions of the world (24). We found that only 12 (10.4%) isolates produce only one toxin (A-B+CDT-), which is lower than what has been reported previously in Beijing (23.3%) (17). Interestingly, infection with toxin-positive *C. difficile* isolates has been associated with higher mortality and recurrence rates (25). In this study, three (2.6%) isolates of *C. difficile* were positive for both the two toxin genes and binary toxin gene (A+B+CDT+), which is similar to that reported by another study in Shanghai (1.6%) (26). However, the overall prevalence of A+B+ *C. difficile* isolates in China is much lower than that in North America and Europe (27). Global variability in the prevalence of *C. difficile* strains with A+B+ could be due to the differences in testing methods, patient-related factors, surveillance sensitivity, and differential infection control practices and distribution of toxigenic *C. difficile* isolates (9).

Based on the MLST analysis, a total of 27 *C. difficile* STs were identified, which were divided into four main clades. The most common four STs (ST3, ST39, ST54, and ST26) were different from our previous study (9). Our previous study on CDI among patients with hospital-acquired pneumonia showed that the predominant STs of *C. difficile* were ST54 (20%), ST37 (15.6%), and ST3 (9.4%) (4). This might be related to the clonal spread of *C. difficile* isolates in the hospital. ST3 and ST54 were the main ST types in *C. difficile* isolates with *tcdA*-positive and *tcdB*-positive (A+B+CDT-). ST37 was the most common ST type in *C. difficile* isolates with only *tcdB*-positive (A-B+CDT-). In another study, ST54 was the most common ST type in *C. difficile* isolates with two toxin genes (tcdA+, tcdB+, cdtB-) (28). Meanwhile, ST37 belonged to *C. difficile* isolates with only *tcdB*-positive (A-B+CDT-) in another study in China (29). *C. difficile* isolates with binary (A+B+CDT+) belonging to ST5 and ST39. All the ST26 strains in this study were NTCD, which is supported by Couturier et al. (30).

Among these 115 toxigenic *C. difficile* isolates, 92 strains (80.0%) were resistant to at least three antibiotics, and multiple resistance to CHL, CLI, TE, AMP, LVX, and FSA (20 strains) were most common, followed by 12 strains were resistant to four antibiotics (CHL+CLI+AMP+FSA) (Figure 3). High-level resistance to AMP, CHL, CLI, and LVX was detected among the isolates with A-B+ and A+B+ in this study. The rates of CLI, TE, and AMP resistance among ST2 *C. difficile* isolates were lower than those in other STs. Previous data showed that resistance to CLI (8.3−100%), cephalosporins (51%), erythromycin (13–100%), and fluoroquinolones (47%) is commonly observed in *C. difficile* isolates within the past 15 years (2000–15) (31). Similar antibiotic resistance was also found in the *C. difficile* isolates investigated in this study. These data in our study suggest that antibiotic resistance of *C. difficile* remains prevailing. More worrisome, most of the *C. difficile* isolates investigated in this study showed resistance to multiple antibiotics.

Although several studies have reported the increasing MICs for MTZ and vancomycin in *C. difficile*, all isolates in the current study were susceptible to these two antibiotics. Both MTZ and vancomycin are the most commonly used antibiotics for mild-to-moderate and severe CDI (32), and they have been used for more than 30 years. In a multicenter study conducted in Taiwan, all *C. difficile* isolates were susceptible to MTZ, however, two isolates had reduced susceptibility to vancomycin (MIC = 4 μg/ml) (33). Furthermore, the MICs of tigecycline and fidaxomicin were low in our study. Fidaxomicin is associated with a significantly lower recurrence rate in CDI therapy, therefore, it is considered to have similar therapeutic efficacy as oral vancomycin (8, 12). In addition to vancomycin, MTZ, and fidaxomicin, tigecycline has been used in cases where severe adverse effects occurred following standard therapy (34). Although *C. difficile* isolates are resistant to multiple antibiotics in our study, antimicrobial therapy is still the first choice for CDI, and specific guideline recommendations of antimicrobial therapy should be based on the severity of the CDI.

## Conclusions

In conclusion, *C. difficile* isolates from CDI patients in our hospital are dominated by those producing two toxins (A+B+CDT-). ST3 isolates are the most prevalent ST, and ST26 isolates are all NTCD. The higher positive rate of *C. difficile* might be due to the wide administration of broad-spectrum antibiotics in recent years. Although multidrug resistance is present in ≥50% of the *C. difficile* isolates, MTZ and vancomycin are still effective against *C. difficile*, serving as available treatment options for CDI patients.

## Data Availability Statement

The original contributions presented in the study are included in the article/supplementary material, further inquiries can be directed to the corresponding authors.

## Author Contributions

Conceptualization and methodology by XM, XH, and AW. Software and formal analysis by ZP and CF. Data collection by CZ, YW, SL, and JD. Writing-original draft preparation by XM. Writing-review and editing by XM, CL, and AW. Funding acquisition by CL and AW. All authors have read and agreed to the published version of the manuscript.

## Funding

This work was supported by the Key Research and Development Projects of Hunan Province (Nos. 2020SK3027 and 2020SK3028) and Natural Science funding of Hunan Province (No. 2021JJ31071).

## Conflict of Interest

The authors declare that the research was conducted in the absence of any commercial or financial relationships that could be construed as a potential conflict of interest.

## Publisher's Note

All claims expressed in this article are solely those of the authors and do not necessarily represent those of their affiliated organizations, or those of the publisher, the editors and the reviewers. Any product that may be evaluated in this article, or claim that may be made by its manufacturer, is not guaranteed or endorsed by the publisher.
